# EFMviz: A COBRA Toolbox Extension to Visualize Elementary Flux Modes in Genome-Scale Metabolic Models

**DOI:** 10.3390/metabo10020066

**Published:** 2020-02-12

**Authors:** Chaitra Sarathy, Martina Kutmon, Michael Lenz, Michiel E. Adriaens, Chris T. Evelo, Ilja C.W. Arts

**Affiliations:** 1 Maastricht Centre for Systems Biology (MaCSBio), Maastricht University, 6229 ER Maastricht, The Netherlands; 2 Department of Bioinformatics—BiGCaT, School of Nutrition and Translational Research in Metabolism (NUTRIM), Maastricht University, 6229 ER Maastricht, The Netherlands; 3 Institute of Organismic and Molecular Evolution, Johannes Gutenberg University Mainz, 55128 Mainz, Germany; 4 Preventive Cardiology and Preventive Medicine—Center for Cardiology, University Medical Center of the Johannes Gutenberg University Mainz, 55131 Mainz, Germany; 5 Department of Epidemiology, CARIM School for Cardiovascular Diseases, Maastricht University, 6229 ER Maastricht, The Netherlands

**Keywords:** elementary flux modes, genome-scale metabolic models, data visualization, network visualization, SBML

## Abstract

Elementary Flux Modes (EFMs) are a tool for constraint-based modeling and metabolic network analysis. However, systematic and automated visualization of EFMs, capable of integrating various data types is still a challenge. In this study, we developed an extension for the widely adopted COBRA Toolbox, EFMviz, for analysis and graphical visualization of EFMs as networks of reactions, metabolites and genes. The analysis workflow offers a platform for EFM visualization to improve EFM interpretability by connecting COBRA toolbox with the network analysis and visualization software Cytoscape. The biological applicability of EFMviz is demonstrated in two use cases on medium (*Escherichia coli*, iAF1260) and large (human, Recon 2.2) genome-scale metabolic models. EFMviz is open-source and integrated into COBRA Toolbox. The analysis workflows used for the two use cases are detailed in the two tutorials provided with EFMviz along with the data used in this study.

## 1. Introduction

Cellular metabolism is comprised of many parallel chemical interactions. Metabolic reactions consume or produce compounds in the cell. Proteins catalyzing these metabolic reactions are called enzymes and the conversion rate of metabolic reactions is termed metabolic flux. Metabolic reactions are concatenated into metabolic pathways which can act simultaneously in the cellular environment, forming highly connected, complex networks. In the past two decades, novel experimental technologies have resulted in an explosion of “omics” data for various organisms which, together with computational advancements, has enabled the construction of large genome-scale metabolic networks. Integration of omics data in such networks opens new avenues to understanding cellular processes in greater detail [1].

A method that has been used to comprehensively study pathways in metabolic networks is Elementary Flux Mode (EFM) analysis [2]. EFMs provide mathematical frameworks to describe non-decomposable steady-state fluxes through metabolic networks [3]. EFM analysis has already been applied in several studies, such as biomass optimization in microorganisms [4], knockout strategy design [5], pathway efficiency [6], strain design [7,8,9], robustness analysis [10] and determining substrate cycles [11]. However, one of the challenges of EFM analysis is the large number of EFMs which grow exponentially with the size of the network [12]. More than two million EFMs have been reported for the metabolic network describing the central metabolism in *Escherichia coli*, which contains merely 110 reactions [13]. In human models, which are by far the largest genome-scale models, the number of EFMs is estimated to be at least 10^29^ [14,15]. Therefore, the field of EFM analysis is driving towards development of better algorithms for computing either the full set of EFMs or a subset of interest. Several algorithms based on linear programming and graph theory have been developed, some of which have even enumerated a subset of EFMs in large human genome-scale models [16,17,18,19,20,21,22,23]. These recent developments have enabled investigation of EFMs from human models to identify, for instance, tissue-specific metabolic pathways and to study differences between cancer subtypes [21,24].

**Figure 1:**
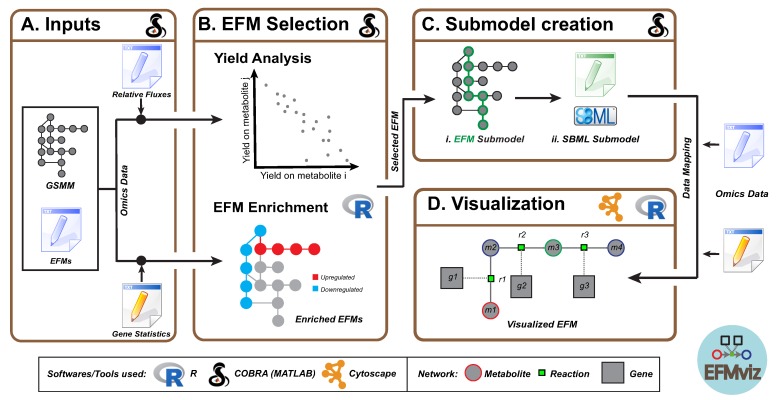
Overview of EFMviz, a new extension of COBRA Toolbox, for Elementary Flux Mode (EFM) visualization. Each box corresponds to a step of the workflow, and the tools/packages used are shown in the top right side of the boxes. (**A**) This workflow takes a Genome-Scale Metabolic Model (GSMM) and a list of EFMs calculated from this model as inputs. (**B**) Data-dependent EFM selection is performed through either yield analysis or EFM enrichment using omics data: in this case, a list of relative fluxes of reactions and differentially expressed genes repectively. (**C**) Once an EFM is selected, a “submodel” is created which is (**D**) visualized in Cytoscape where other omics data can also be mapped.

Previous studies have combined network visualization with optimization methods based on genome-scale metabolic models (Flux Balance Analysis (FBA) [25]), such as the Escher platform [26], FluxViz [27] for FASIMU [28], FluxMap and FBASimVis plugins for VANTED [29], Paint4Net [30] extension in COBRA Toolbox [31], CellNetAnalyzer [32], MetDraw [33], ReconMap [34] and FluxVisualizer [35]. Most of these approaches only support flux visualization (experimental or simulated), but it is not possible to integrate other quantitative omics data for the molecules in the network. They generate static maps (Paint4Net) or they are available within application-specific standalone tools (VANTED, FASIMU). While MetExploreViz [36] offers a solution for mapping omics data on metabolic networks, it is not designed for use with EFMs. Although the interactive web application, Escher, supports overlaying FBA results and omics data, it uses static maps and supports visualization of up to 200 reactions. Specifically, in the context of EFM analysis, the efforts on network visualization have been limited [37] since the focus has been on algorithm improvement. Some EFM tools do provide a basic visualization but have limited capabilities for data mapping and integration. Therefore, in this paper we present a new extension of COBRA Toolbox to facilitate the analysis, selection and visualization of generated EFMs (Figure 1). EFMviz can use the EFMs generated from any algorithm and aims to enhance the interpretability of EFMs. Towards this goal, EFMviz provides functionality to (1) select relevant EFMs based on *yield analysis* and *EFM enrichment*, (2) compare EFMs using preserved visual arrangement and *subsystem occurrence*, (3) assess overlap between EFMs (*backbone identification*) and (4) construct advanced visualizations including omics data mapping on reactions and molecules. We demonstrate the applicability of EFMviz using two genome-scale models: (1) a medium-sized, *E. coli* model (iAF1260, reference [38]) and (2) a large, human model (Recon 2.2, reference [39]). The analysis workflows used for the two use cases are detailed in the two tutorials provided with EFMviz.

## 2. Materials and Methods

The highlight of EFMviz is the visualization of an EFM as a network of reactions, metabolites and genes. The workflow consists of seven MATLAB functions: *efmImport, efmFilter, efmYieldAnalysis, efmEnrichmentAnalysis, efmSubmodelExtractionAsSBML, efmSubsystemsExtraction* and *efmBackboneExtraction*. Additionally, a tutorial accompanies each use case which walks the user through all the steps necessary to not only reproduce results from this study but also to adapt other EFMs of interest. This section describes each component of the workflow in detail (Figure 1). All the use-case-specific modifications to the workflow will be explained in Results.

Genome-scale metabolic models contain genes, metabolites, reactions and their relationships, including the stoichiometric matrix. These components are organized into various compartments corresponding to intracellular organelles [40]. The COBRA toolbox is a standard MATLAB software suite for modeling and analysis of genome-scale models and was also used for model manipulation in the present workflow (Figure 1).

### 2.1. Inputs

This workflow takes a file containing EFMs along with the genome-scale metabolic model used for EFM calculation as inputs (Figure 1A). EFMs generated from any tool are compatible with the workflow. Each row in the (space delimited) text file with EFMs should contain the reaction indices of reactions active in an EFM and the workflow supports a COBRA model format. Optionally, relative fluxes of reactions active in an EFM, which are also computed during EFM calculation by most tools, and/or omics data, can also be used for selecting EFMs for visualization. In this study, EFMs were computed using TreeEFM [41] (see Supplementary Information for details on EFM Generation). Additionally, relative fluxes (in Use Case 1) and gene level statistics (from differential expression analysis, in Use Case 2) were used as inputs in this study. In the first step, EFMs were imported into MATLAB for the current analysis along with the corresponding model and use case specific data (Figure 1A).

### 2.2. EFM Selection

Previous studies have reported a large number of EFMs for a genome-scale metabolic model. The length of the EFMs can also vary greatly, ranging from very small with less than 10 reactions to very large with more than 1000 reactions. To facilitate better understanding and visualization of such complex and overwhelming EFMs, a selection criterion can be applied to analyze EFMs, depending on the biological phenomenon under study. EFMs containing a reaction of interest can be retained; for example, glucose-uptake-EFMs that also release ethanol or lysine-releasing-EFMs that also take up glucose. Data-related features can also be determined at this stage, using omics data, such as transcriptomics, fluxomics or proteomics, if available. These features can be used to filter and select EFMs for visualization; for instance, EFMs with the highest number of differentially expressed genes. To investigate the metabolic diversity in the filtered EFMs and to select EFMs for visualization, two approaches were used in this study for EFM Selection, yield analysis (in Use Case 1) and EFM enrichment (in Use Case 2) (Figure 1B).

#### 2.2.1. Yield Analysis

The concept of “yield analysis” was first introduced by Song and Ramkrishna [42] as a method of extracting a subset of EFMs describing a particular metabolic behavior, thereby reducing the vast amount of EFMs. A crucial step for calculation of yield is the choice of input and output reactions of interest, i.e., uptake of a substrate and excretion of a product, following which yield is simply calculated by dividing the output flux by the reference input flux [42].

#### 2.2.2. EFM Enrichment

Statistical enrichment analysis enables identifying EFMs characterizing a biological condition. Rezola et al. [21] used a multivariate hypergeometric test to distinguish tissue-specific EFMs amongst EFMs calculated from generic human metabolic network. For the present analysis, an EFM was considered as a set of reactions. Gene level statistics, typically obtained after differential gene expression analysis, were mapped on these reactions using GPR rules of each reaction as defined in the model. If a reaction was catalyzed by genes A and B with the GPR of “gene A AND gene B”, then the p-value and log2 fold change of the gene with minimum expression were assigned to the reaction. On the other hand, if the GPR was “gene A OR gene B”, then the p-value and log2 fold change of the gene with maximum expression was assigned to the reaction. Data processing for enrichment was performed in MATLAB, and the enrichment itself was carried out in an R script (R version 3.5.1, reference [43]) using the package Piano [44] (Figure 1B). Using an enrichment analysis, reaction sets (or EFMs in this case) enriched with upregulated or downregulated genes can be identified, which in turn would provide insights into how the changes in gene expression might be translated to the metabolic level.

Using the above mentioned criteria, we highlight how an EFM can be selected for further analysis through visualization; for instance, EFM with minimum yield or maximum yield or the most significantly enriched EFM.

### 2.3. Submodel Creation

Once an EFM was selected, the reactions in this EFM were identified using the model that was previously imported. All the remaining reactions were removed from the model, along with any unconnected genes and metabolites, thereby creating a “submodel” (Figure 1Ci). This submodel was saved as an SBML file (Figure 1Cii) which was then visualized and analyzed in Cytoscape [45]. Using the workflow presented here, individual submodels can be created for multiple EFMs, satisfying various selection criteria mentioned above, and can be visualized within a single Cytoscape session (Figure S4).

### 2.4. Visualization

Cytoscape is an open-source tool commonly used for visualization and analysis of biological networks. It not only enables data visualization and advanced network analysis, but also network extension with other types of molecular data, all within a single framework. Additional functionality can be accessed through various apps that can easily be installed within the Cytoscape GUI. Cy3sbml [46] is one such app that has exclusively been designed to handle SBML model files in Cytoscape. It enables network visualization using the SBGN standard [47], where each type of molecular species (metabolites, genes and reactions) is assigned a node type with the node border being color coded according to cellular compartment. For the present analysis, Cytoscape version 3.6.1 and cy3sbml version 0.2.7 were used. In order to visualize the selected EFM, the submodel that was generated beforehand as an SBML file was opened in Cytoscape using cy3sbml (Figure 1D). Three networks were generated for every model imported and they are respectively identified using the prefixes: All, Base and Kinetic. The Base network was chosen for further investigation. Cytoscape allows application of automatic layout algorithms based on the yFiles diagramming libraries [48] for arranging the graphical components. yFiles layout algorithms are available for Cytoscape (v3.6.0 and above) under a license agreement and yFiles app version 1.0.1 was installed and used. To facilitate interpretation of the visualized EFM and minimize manual effort on the graphical arrangement, the yFiles Orthogonal Layout was applied on the Base network. At this stage, the data that were used during EFM selection, reaction fluxes (Use Case 1) and gene expression (Use Case 2), were also imported into Cytoscape and mapped on the visualized EFM. All the data manipulations (such as identifier matching, necessary for mapping) and network operations described above, except layout application, were automated through an R script (R version 3.5.1) using the library, RCy3 [49]. The use cases presented here demonstrate mapping of two different types of data on the visualized EFMs. NDEx [50] links for all the EFMs visualized in this study have also been provided for further interactive exploration. When EFMs contain ubiquitous metabolites, such as ATP, H^+^ and H_2_O, the visualized network can be difficult to interpret due to their participation in numerous reactions. In such cases, these metabolites can be removed from visualization during submodel creation. In the present work, the EFMs without ubiquitous metabolites have been visualized and are described in Results, and the corresponding networks with ubiquitous metabolites are provided in the Supplementary Information along with the list of removed metabolites.

## 3. Results

This section describes two biological applications of EFMviz on two different models, iAF1260 (*E. coli*) and Recon 2.2 (human).

### 3.1. Use Case 1: Visualizing Elementary Flux Modes from the E. coli Model

The first use case demonstrates our visualization workflow on *E. coli* which has been widely used to produce industrially important compounds such as recombinant proteins and biofuels [51,52]. However, a high growth rate is accompanied by acetate production, which inhibits growth, diverting carbon from biomass formation and is detrimental to product synthesis [53,54]. Here, we aim to showcase an application of EFMviz using acetate-releasing EFMs and yield analysis which can be used as means of studying acetate overflow metabolism.

#### 3.1.1. Model

For this analysis, the model iAF1260 [38], was used. It contains 1261 genes, 1668 metabolites and 2382 reactions.

#### 3.1.2. Analysis

**Figure 2:**
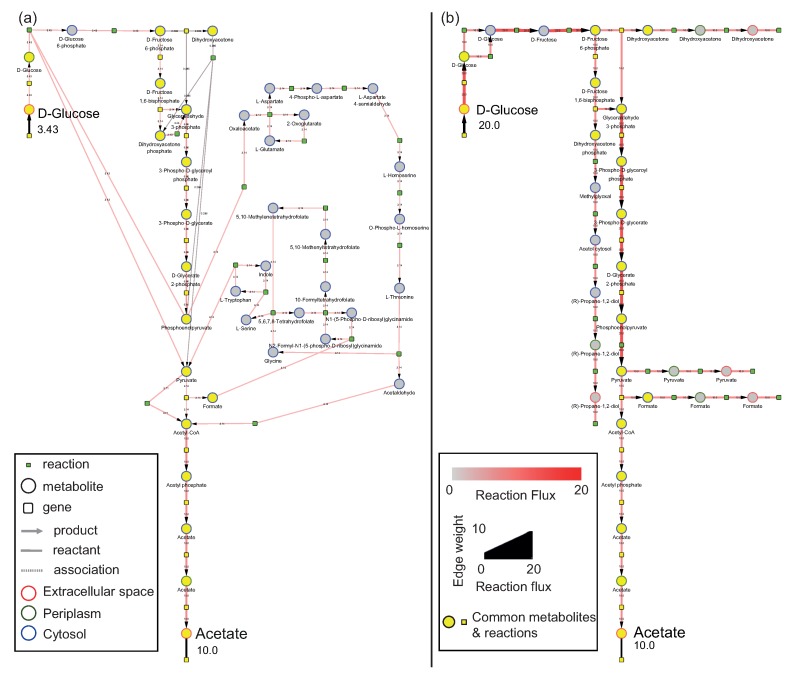
The EFMs from iAF1260 with (**a**) the highest and (**b**) lowest acetate yields on glucose. The uptake and release reactions are highlighted in black. Metabolite node colors indicate the intracellular location, extracellular space (red), cytosol (blue) and peroxisome (green). Relative reaction fluxes, as obtained from EFM calculations, have been mapped on the edges in both the networks. Metabolites and reactions common to both networks are highlighted in yellow. The NDEx links http://bit.ly/2MEQ0v7 and http://bit.ly/2QIrV8f can be used to study the networks interactively.

EFMs that were generated for the acetate exchange reaction, EX_ac_e, (see Supplementary Information) were loaded into MATLAB along with relative fluxes that were also generated during EFM calculation. A total of 948 EFMs were imported, and all the EFMs also contained the glucose uptake reaction, EX_glc_D_e_b. Yield was calculated using glucose uptake and acetate release as the input and output respectively. Among the EFMs under consideration, the relative fluxes of glucose uptake ranged from 3.43 to 20 mmol gDW^−1^ h^−1^. Values for yield ranged from 0.5 to 2.9. The EFMs with both highest (one EFM, EFM 18) and lowest yields (84 EFMs) were identified. EFM 18 and the shortest EFM with lowest yield (EFM 885) were selected for visualization. A submodel corresponding to each of the selected EFMs was generated without ubiquitous metabolites and visualized in Cytoscape (Figure 2a,b) respectively. The same EFMs with ubiquitous metabolites have been visualized in Figures S1 and S2. Additionally, reaction fluxes from the respective EFMs were mapped on the visualized networks. If available, experimental or simulated fluxes can also be mapped on the EFMs in place of relative fluxes. Both networks show two types of nodes, metabolite as circles and reactions as small green squares. Genes/enzymes associated with each reaction can also be visualized; however, since the focus was on reaction fluxes for Use Case 1, genes have been hidden post visualization in Figure 2a,b. Border color on metabolite nodes corresponds to intracellular location: extracellular space (in red), cytosol (in blue) and periplasm (in green). Edge thickness represents the amount of reaction flux which is also numerically indicated on the edges.

##### Comparison of EFMs Using Preserved Visual Arrangement

As mentioned earlier, this workflow can be applied to several EFMs at a time. A useful feature for visualizing and understanding multiple networks is having the shared nodes at the same position across networks. Such a preserved arrangement facilitates visual comparison by highlighting the differences between EFMs immediately. Using the utility of Copycat Layout in Cytoscape, the node positions of Figure 2a were used to lay out the nodes in Figure 2b, thereby achieving a conserved graphical arrangement between the two EFMs. With such a layout, it can be seen that the processes resulting in excess yield revolve around phosphoenolpyruvate, pyruvate and acetyl-CoA (Figure 2a). EFMs shown in Figure 2a,b contain 37 and 35 reactions respectively, of which 15 reactions are common. Although both networks release 10 gDW^−1^ h^−1^ of acetate, an increased amount of glucose is taken up in Figure 2b (20 gDW^−1^ h^−1^) compared to only 3.43 gDW^−1^ h^−1^ in Figure 2a. Taking a closer look into the processes, it can be observed that formate and pyruvate are released in Figure 2b but are used by glycine and serine metabolism in Figure 2a. Following a series of reactions, this diversion ultimately results in an increased flux towards acetyl-CoA despite taking up less glucose. In addition, pyruvate is the only source of acetyl-CoA production in Figure 2b, whereas, Figure 2a shows an additional route from acetaldehyde. In summary, the high acetate yield stems from a combination of processes involving pyruvate, formate and acetaldehyde.

### 3.2. Use Case 2: Visualizing Elementary Flux Modes from the Human Model

The second use case demonstrates EFMviz using lactate-releasing EFMs and EFM enrichment as a means of studying Warburg effect [55].

#### 3.2.1. Data

The publicly available RNA sequencing data from the TCGA breast cancer project was used for this analysis [56]. Using the edgeR package in R [57], the RNA-sequencing count data was preprocessed, and lowly expressed genes were excluded. Additionally, differential analysis between the tumor and healthy tissue was performed with edgeR. These differentially expressed genes were used for EFM enrichment in the next step (Figure 1B) and were also mapped on the visualized EFM network.

#### 3.2.2. Model

For this analysis, the human reconstruction, Recon 2.2, was used. The COBRA model contains 1675 genes, 5324 metabolites and 7785 reactions.

#### 3.2.3. Analysis

EFMs that were generated for the glucose uptake reaction, EX_glc(e)_b, (see Supplementary Information) were loaded into MATLAB. A total of 84 EFMs were imported, among which those containing the lactate release reaction, EX_lac_L(e)_f were retained for further analysis. Statistical enrichment analysis was performed using differential gene expression to understand metabolic differences in the EFMs which describe processes involving glucose uptake and lactate release. EFM enrichment analysis resulted in 17 filtered EFMs that were significant (p< 0.05, FDR adjusted) and upregulated in cancer.

##### Comparing EFMs Using Subsystem Occurrence

**Figure 3:**
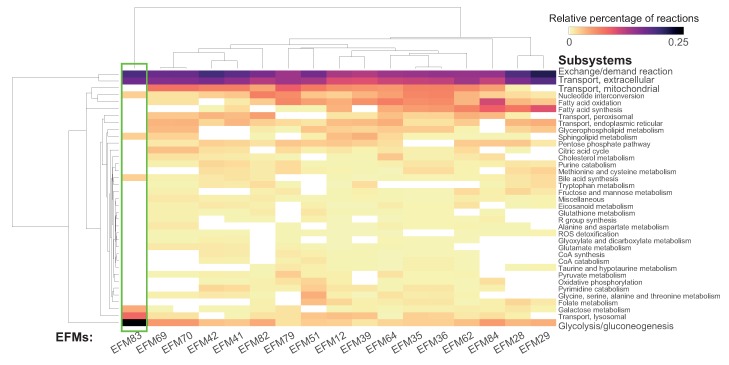
Subsystem occurrence in the significantly enriched EFMs indicating distribution of reactions across different subsystems. Seventeen of the EFMs that take up glucose and release lactate, were found to be significantly upregulated in cancer. The subsystems in the EFM selected for visualization are highlighted in the green rectangle.

**Figure 4:**
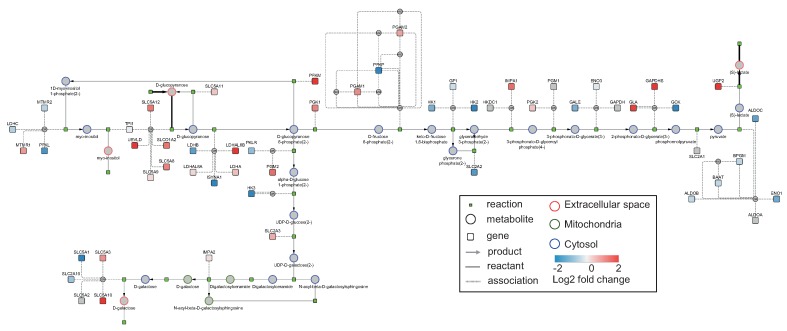
EFM from Recon 2.2 that takes up glucose and releases lactate. This EFM had the highest density of reactions in glycolysis/gluconeogenesis and galactose metabolism. The uptake and release reactions are highlighted in black. Metabolite node colors indicate the intracellular location, extracellular space (red), cytosol (blue) and mitochondria (green). Changes in gene expression between breast cancer tumor and healthy tissue (log2 fold change) have been mapped on the genes. Using this NDEx link, http://bit.ly/39tf1Ub, the network can be studied interactively.

We further probed into “subsystem occurrence”, i.e., the distribution of reactions across subsystems, to understand the differences in the processes among the significantly enriched EFMs. The reactions in all of the 17 significantly enriched EFMs belonged to a total of 37 subsystems and were predominantly involved in transport, central carbon metabolism, fatty acid metabolism and nucleotide interconversion (Figure 3). About 15–25% of reactions in all the EFMs are involved in exchange/demand and extracellular transport. Reactions participating in glycolysis/gluconeogenesis were found in all the EFMs. Other processes from the central carbon metabolism contained a low distribution of reactions (<10%). In terms of overlap of reactions between the enriched EFMs, no clear clusters could be identified between EFMs (as seen in the column-wise dendrogram in (Figure 3)) indicating that they are quite divergent. However, five pairs of EFMs, 1 (EFM 69 and EFM 70), 2 (EFM 41 and EFM 42), 3 (EFM 12 and EFM 39), 4 (EFM 35 and EFM 36) and 5 (EFM 28 and EFM 29), showed high similarity in terms of processes, among which the pairs 1 and 2 even differed by a single reaction. Interestingly, despite being the shortest EFM with 41 reactions, EFM83 (p adjusted = 0.002), highlighted in green in Figure 3, stands out due to its highest density of reactions in glycolysis/gluconeogenesis and galactose metabolism, and thus, was selected for visualization. A submodel corresponding to the selected EFM was generated without ubiquitous metabolites and visualized (Figure 4). The same EFM with ubiquitous metabolites has been visualized in Figure S3. Gene expression data was mapped on the visualized network. This network shows three type of nodes, metabolite as circles, reactions as small green squares and genes as large squares. Border color on metabolite nodes corresponds to intracellular location: extracellular space (red), cytosol (blue) and mitochondria (green). Figure 4 contains more upregulated genes (25 out of 55) than downregulated genes (23 out of 55).

### 3.3. Assessing Overlap between EFMs: Backbone Identification

**Figure 5:**
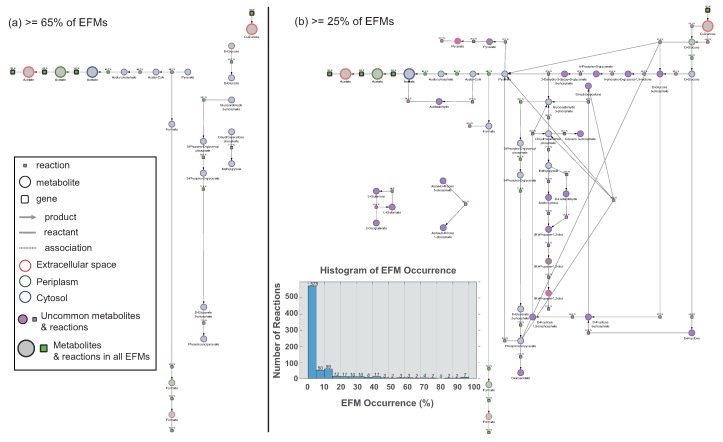
Backbone reactions present in (**a**) greater than 65% and (**b**) greater than 25% of acetate-release EFMs from *E. coli*. Metabolite node colors indicate the intracellular location, extracellular space (red), cytosol (blue) and periplasm (green). Metabolites and reactions that are different between the two networks are highlighted in pink. Inset shows the distribution of reactions occurring in different proportions of EFMs. The NDEx links http://bit.ly/2SCruPi and http://bit.ly/2Q6ry8i can be used to study the networks interactively.

Apart from visualizing an EFM, a “backbone” can also be extracted and visualized from any given set of EFMs. We define *backbone* as reactions that are present in a large number (80–90%) of EFMs. A visual comparison of such a backbone, from different proportions of EFMs, reveals the most commonly occurring reactions and gives an indication of the underlying conserved structure. Through this added functionality, the backbone reactions present in 65% and 25% of EFMs were extracted from the EFMs from *E. coli* (Figure 5a,b). Ubiquitous metabolites have been removed from both the networks and genes were hidden post visualization. Copycat Layout was applied to both EFMs. The proportion of EFMs any given reaction is found in, is indicated by a percentage value on every reaction node. The metabolites and reactions occurring in all the EFMs are enlarged slightly. To facilitate comparison of processes across EFM proportions, uncommon nodes are highlighted in pink. In the inset, it can be seen that most reactions are present in fewer EFMs and only 11 reactions are present in more than 80% of EFMs. Low numbers of reactions at high cut-off values result in a disconnected network (Figure 5a). As found earlier, glucose uptake and acetate release reactions were present in all the EFMs, and this is also seen in both the networks. With a reduction in the proportion of EFMs, the size of the backbone increases and connections develop between the seemingly disconnected nodes (Figure 5b). Though not fully connected, Figure 5b shows reactions involved in glycolysis (pink nodes) connecting the uptake and release processes. The potential of this backbone identification lies in identifying alternative reactions or routes between metabolites. For instance, this set of EFMs contains only two ways of transporting glucose from extracellular space to periplasm, which are seen in 54.1% and 45.9% of EFMs exclusively. On the other hand, conversion of acetyl-CoA to acetate occurs either via acetyl phosphate (85.2%) or acetaldehyde (27%). This implies that these two routes are not exclusive and can occur simultaneously, an example of which was also seen in Figure 3a. Thus, the concept of backbone can be applied on any set of EFMs to draw a complete map of possible routes, starting from uptake to release.

The integrated visualizations of genes, metabolites and reactions within a single network are shown in three different EFMs (Figure 2, Figure 4 and Figure 5). Encoding the three molecular types as different node shapes distinguishes between individual network components. Highlighting both uptake and release reactions with black lines not only demarcates the exchange reactions from reaction-metabolite and gene-reaction interactions in gray lines, but also guides tracing the network from uptake to release. Indicating intracellular compartments by different colors aids in easily identifying where the processes occur within the cell. Larger EFMs generally contain reactions from several subprocesses and could aid in identifying connections between processes beyond classical pathway definitions. Additional data mapping immediately draws the researcher’s attention towards changes in flux or changes in gene expression and could indicate how these changes may propagate through the network. Through mapping two different data types, Figure 2, Figure 4 and Figure 5 illustrate the potential of EFMviz in multiple data integration with EFMs. In summary, the features described above enable an integrated visualization in terms of both network components and data mapping.

## 4. Discussion

EFMviz, an extension of COBRA Toolbox, demonstrates graphical visualization of EFMs, which has remained a challenge thus far. In particular, the workflow is compatible with tools used for EFM generation, thereby allowing visualization of EFMs generated from different methods. Applying the workflow on two models of different sizes also shows that this visualization framework is not restricted by model size. Through various aspects of visualization described here, the importance of better visualization strategies in assisting human cognition is emphasized. The network modification aspects of Cytoscape were exploited to map experimental and simulated data on the visualized networks, thereby providing a quantitative context to the qualitative EFMs. Two different types of data were successfully mapped on the visualized EFMs, (a) relative reaction fluxes (in Use Cases 1) and (b) differential expression of genes (in Use Case 2). Nevertheless, other molecular data, such as metabolomics, proteomics and C13 fluxes, can also be mapped effortlessly.

In addition, the approach presented here facilitates both the automation of network operations in Cytoscape and the exploration of large EFMs. Due to yFiles libraries being available only in the Cytoscape application, layout application was performed using the GUI. Nevertheless, automation of network manipulation minimizes both user effort and time, thereby accelerating EFM investigation. EFMviz also allows simultaneous exploration of multiple EFMs within a single Cytoscape session. Supplementary Figure S4 shows a Cytoscape session with six different EFMs (four from Use Case 1 and two from Use Case 2) visualized in a single Cytoscape session. The NDEx links of the networks provided here allow the readers to interactively explore the networks. The advantage of Copycat Layout lies not only in better visual comparison but also in identification of alternative routes between metabolites common to networks under comparison; for example, different reactions connect glucose to pyruvate and pyruvate to acetyl-CoA (Figure 2).

Every EFM imported into MATLAB can be visualized using EFMviz. However, trying to navigate through and understand several networks simultaneously, even 10–15, can be very difficult to comprehend. Therefore, it is highly recommended to apply a series of filtering criteria (as demonstrated in the use cases) in order to select the EFM(s) most relevant for the research question under study. EFMviz is currently limited to the MATLAB implementation of COBRA toolbox, but an EFMviz extension for COBRApy (Python) is planned to support a broader community.

Apart from the advanced visualizations and data mapping, EFMviz offers various strategies for both selecting relevant EFMs and comparing EFMs. Thus, the standardized integrative workflow presented here marks an important step forward for EFM analysis. It is important to note that the visualization aspect of EFMviz is not limited to EFMs. In principle, one can visualize any set of reactions, such as (a) a subsystem, (b) a solution of a flux balance analysis (c) or any flux vector, to name a few. Combining metabolic networks with multi omics data, such an integrated visualization approach will serve as an extremely useful tool for characterizing cellular metabolism. Utilizing the powerful, open-source Cytoscape platform not only allows network visualization and data mapping, but also opens new avenues for EFM extension with other molecular components. Taken together, we demonstrate that with effective visualization of the metabolic networks, the proposed workflow can allow for a comprehensive understanding of the biological processes under investigation.

## Supplementary Materials

All the scripts used in this analysis are available at https://github.com/macsbio/efmviz. The following are available online at https://www.mdpi.com/2218-1989/10/2/66/s1. Figure S1: The EFM from iAF1260 with highest acetate yield on glucose. Same network as Figure 2a but with ubiquitous molecules; Figure S2: The EFM from iAF1260 with lowest acetate yield on glucose. Same network as Figure 2b but with ubiquitous molecules; Figure S3: EFM from Recon 2.2 that takes up glucose and releases lactate. Same network as Figure 4 but with ubiquitous molecules; Figure S4: Six EFMs visualized in a single Cytoscape session.
