# Supplementary material for: EFMviz: A COBRA Toolbox Extension to Visualize Elementary Flux Modes in Genome-Scale Metabolic Models
Source: Metabolites. 2020 Feb 12;10(2):66. doi: 10.3390/metabo10020066 (PMC7074156; doi:10.3390/metabo10020066)
Supplement: Supplementary file 1 [file metabolites-10-00066-s001.pdf]

EFMviz: A COBRA Toolbox extension to visualize Elementary Flux Modes in Genome-Scale Metabolic Models

**Chaitra Sarathy [1,*] , Martina Kutmon [1,2] , Michael Lenz [1,3,4] , Michiel E. Adriaens [1] ,
Chris T. Evelo [1,2] and Ilja C.W. Arts [1,5]**

[1] Maastricht Centre for Systems Biology (MaCSBio), Maastricht University, 6229 ER, The Netherlands
[2] Department of Bioinformatics—BiGCaT, School of Nutrition and Translational Research in Metabolism (NUTRIM), Maastricht University, 6229 ER, The Netherlands
[3] Institute of Organismic and Molecular Evolution, Johannes Gutenberg University Mainz, 55128, Germany
[4] Preventive Cardiology and Preventive Medicine—Center for Cardiology, University Medical Center of the Johannes Gutenberg University Mainz, 55131, Germany
[5] Department of Epidemiology, CARIM School for Cardiovascular Diseases, Maastricht University, 6229 ER, The Netherlands
***** Correspondence: chaitra.sarathy@maastrichtuniversity.nl

Supplementary Information

Supplementary Methods

Since TreeEFM (Pey et al., 2015) was used for EFM calculation, both the models were preprocessed in MATLAB before generating EFMs. A necessary starting point in many EFM approaches is to decompose the reversible reactions into forward and backward reactions, so that each reaction carries a non-negative flux (Schuster & Hilgetag, 1994 , de Figueiredo et al., 2009 , Rezola et al., 2013). The models were loaded into MATLAB and were converted to models containing only unidirectional reactions using COBRA Toolbox. The stoichiometric matrix of the irreversible models was saved as a comma separated text file and was used as the input for generating EFMs.

TreeEFM is one of the approaches to generate a subset of EFMs. It is available as a stand-alone software, implemented in C++ and interacts with open-source linear simplex solver, COIN Linear Program (CLP), by the COIN-OR project (Lougee-Heimer, 2003). Given the ability of MATLAB to execute system commands, the call to the stand-alone TreeEFM was also integrated within the MATLAB environment where the model was preprocessed. A key requirement of TreeEFM is the choice of an active reaction, indicating its presence in all the resulting EFMs. Depending on the model and the biological process under study, one reaction must be chosen as the input to TreeEFM, along with the stoichiometric matrix file of the preprocessed model. Active reactions were acetate release EX_ac_e for Use Case 1 and EX_glc(e)_b for Use Case 2. With these inputs, TreeEFM was executed with a stop condition of 10,000 EFMs. Other optional flags included were: (1) mode of tree exploration: deterministic (default) (2) zero threshold (-z): $1.0e^{-6}$ (3) disk caching dropped when free RAM is below (-d drop): 100% (of the total RAM). TreeEFM outputs two files, (1) a (space separated) text file containing all the calculated EFMs where each row has the indices of all the reactions active in the corresponding EFM and (2) a (space

separated) text file containing relative fluxes of all the reactions in an EFM. Both the files were used for analysis in Use Case 1, whereas only the EFMs were used for Use Case 2.

Supplementary Results

Use Case 1:

<u>*List of ubiquitous molecules:*</u> h, h2, fe3, fe2, h2o, na1, atp, datp, hco3, pi, adp, dadp, nadp, nadph, coa, o2, nad, nadh, ppi, pppi, amp, so4, fad, fadh2, udp, dudp, co2, h2o2, nh4, ctp, utp, gtp, cdp, gdp, dcdp, dgdp, dtdp, dctp, dttp, dutp, dgtp, cmp, gmp, ump, dcmp, dtmp, dgmp, dump, damp, cl, q8, q8h2, 2dmmq8.

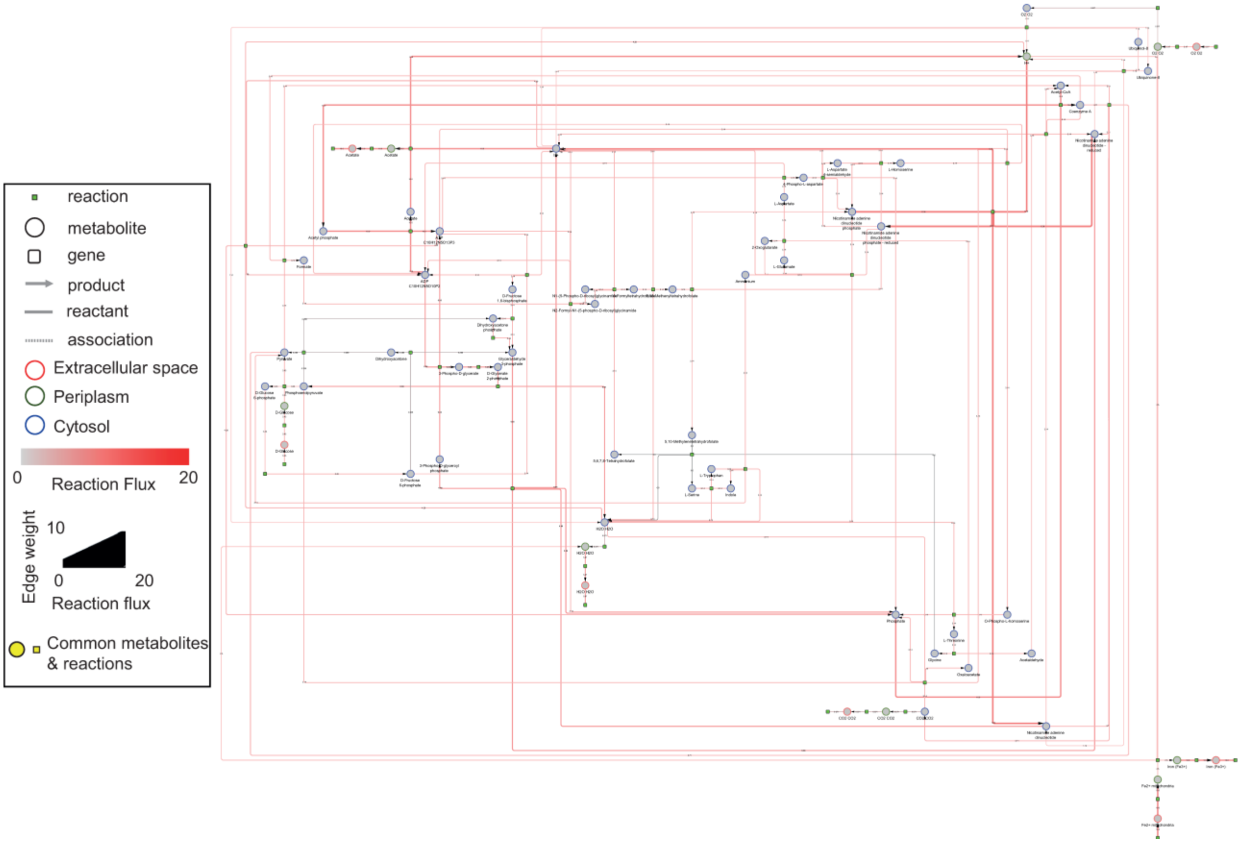

Figure S1: The EFM from iAF1260 with highest acetate yield on glucose. Same network as Figure 2a but with ubiquitous molecules. The uptake and release reactions are highlighted in black. Metabolite node colors indicate the intracellular location, extracellular space (red), cytosol (blue) and peroxisome (green). Relative reaction fluxes, as obtained from EFM calculation have been mapped on the edges in the network. Using this NDEx link, http://bit.ly/2F6tneY, the network can be studied interactively.

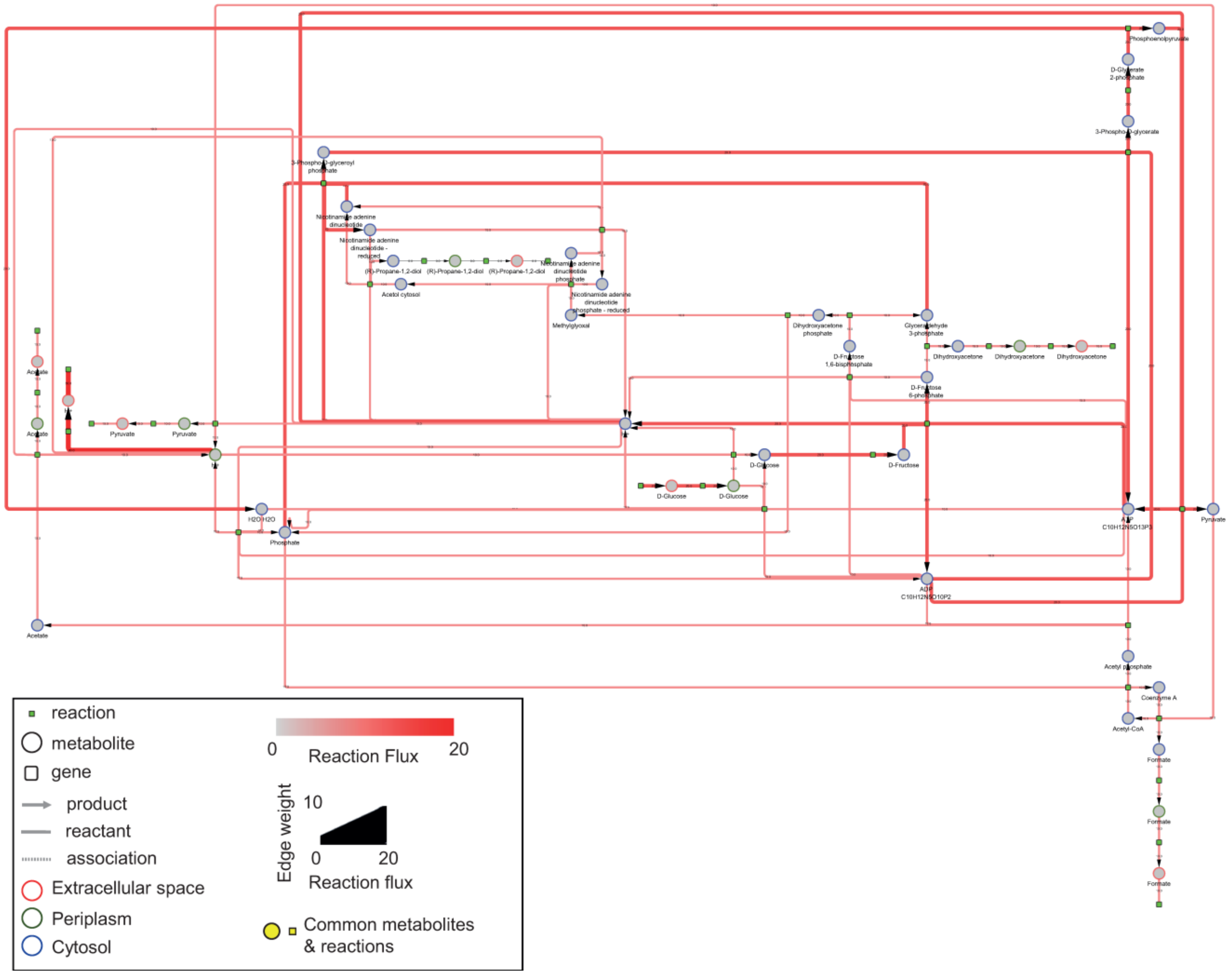

Figure S2: The EFM from iAF1260 with lowest acetate yield on glucose. Same network as Figure 2b but with ubiquitous molecules. The uptake and release reactions are highlighted in black. Metabolite node colors indicate the intracellular location, extracellular space (red), cytosol (blue) and peroxisome (green). Relative reaction fluxes, as obtained from EFM calculation, have been mapped on the edges in the network. Using this NDEx link, http://bit.ly/2Szv2ln, the network can be studied interactively.

Use Case 2:

List of ubiquitous molecules: h, h2, h2o, atp, pi, adp, nad, ppi, pppi, nadh, nadp, nadph, amp, co2, coa, nh4, o2, fad, fadh2, fe2, fe3, na1, gdp, gtp, udp, ctp, utp, cdp, fmn, fmnh2.

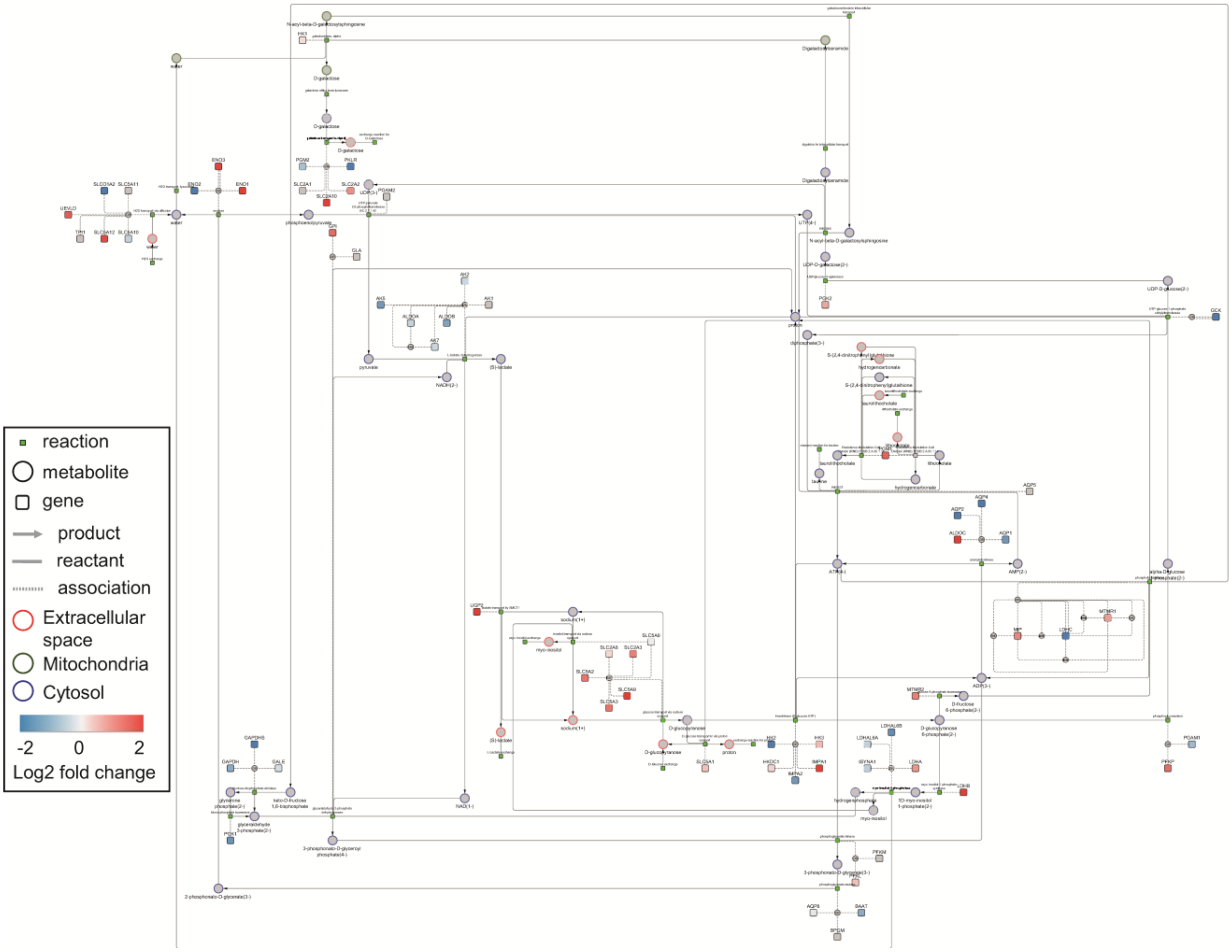

Figure S3: EFM from Recon 2.2 that takes up glucose and releases lactate. Same network as Figure 4 but with ubiquitous molecules. This EFM had the highest density of reactions in glycolysis/gluconeogenesis and galactose metabolism. The uptake and release reactions are highlighted in black. Metabolite node colors indicate the intracellular location, extracellular space (red), cytosol (blue) and mitochondria (green). Changes in gene expression between breast cancer tumor and healthy tissue (log2 fold change) have been mapped on the genes. Using this NDEx link, http://bit.ly/2Uu5w1G, the network can be studied interactively.

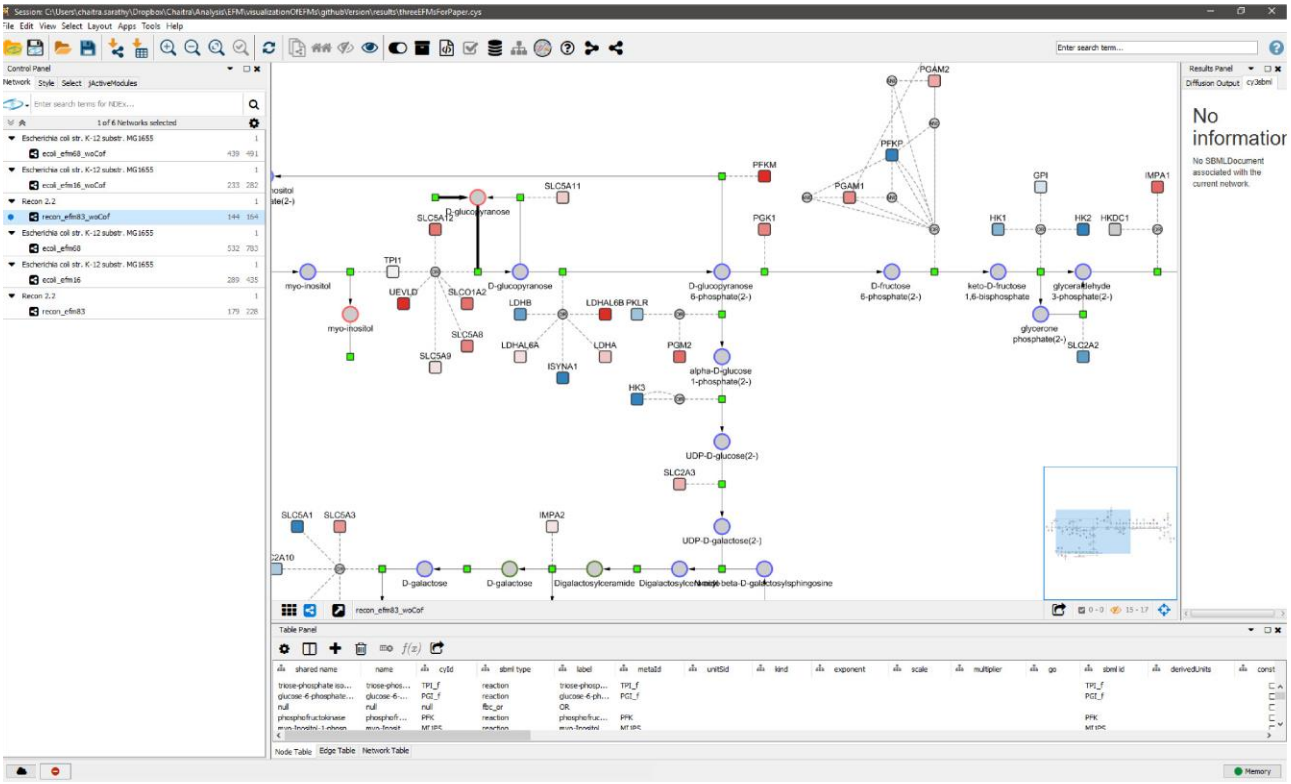

Figure S4: Six EFMs visualized in a single Cytoscape session which can be seen in the panel on the left. Upon selecting one network from the left panel, the EFM is displayed on the right panel.

References

de Figueiredo, L. F., Podhorski, A., Rubio, A., Kaleta, C., Beasley, J. E., Schuster, S., & Planes, F. J. (2009). Computing the shortest elementary flux modes in genome-scale metabolic networks. *Bioinformatics*, *25*(23), 3158–3165. https://doi.org/10.1093/bioinformatics/btp564

Lougee-Heimer, R. (2003). The Common Optimization INterface for Operations Research: Promoting open-source software in the operations research community. *IBM Journal of Research and Development*, *47*(1), 57–66. https://doi.org/10.1147/rd.471.0057

Pey, J., Villar, J. A., Tobalina, L., Rezola, A., García, J. M., Beasley, J. E., & Planes, F. J. (2015). TreeEFM: calculating elementary flux modes using linear optimization in a tree-based algorithm. *Bioinformatics*, *31*(6), 897–904. https://doi.org/10.1093/bioinformatics/btu733

Rezola, A., Pey, J., de Figueiredo, L. F., Podhorski, A., Schuster, S., Rubio, A., & Planes, F. J. (2013). Selection of human tissue-specific elementary flux modes using gene expression data. *Bioinformatics*, *29*(16), 2009–2016. https://doi.org/10.1093/bioinformatics/btt328

Schuster, S., & Hilgetag, C. (1994). On Elementary Flux Modes In Biochemical Reaction Systems at steady state. *Journal of Biological Systems*, *02*(02), 165–182. https://doi.org/10.1142/S0218339094000131
